# Mechanical mechanism of suture passer needle break in rotator cuff repair

**DOI:** 10.3389/fsurg.2022.1065666

**Published:** 2022-12-19

**Authors:** Chunxi Yang, Cheng Xie, Hui Liu, Zikai Hua, Bingchen An

**Affiliations:** ^1^ Department of Bone and Joint Surgery, Department of Orthopedic, Renji Hospital, Shanghai Jiao Tong University School of Medicine, Shanghai, China; ^2^ Department of Rehabilitation Medicine, Huadong Hospital, Fudan University, Shanghai, China; ^3^ The second rehabilitation Hospital of Shanghai, Shanghai, China; ^4^ Orthotek Laboratory ACAD, School of Mechatronics Engineering and Automation, Shanghai University, Shanghai, China

**Keywords:** rotator cuff, arthroscopic surgery, suture passing device, broken needle, mechanical mechanism

## Abstract

**Introduction:**

Suture passer needle, as one of commonly used instrument in the arthroscopic rotator cuff repair, often breaks at the notch of the needle, which originally was designed to facilitate suture with thread. Our study aimed to evaluate the suture failure rate and stitch success rate between intact suture needle and broken needle and explore the mechanism of the needle breakage and achieving better future designs.

**Materials and methods:**

From 2017 to 2021, consecutive 437 shoulders (11 cases were bilateral) underwent arthroscopic repair for full-thickness rotator cuff tear at the authors’ institution. The breakage of needles was recorded. Finite elements analysis and mechanical test were utilized to compare stress distribution, puncture performance, and loaded puncture performance between the broken needle and the intact needle.

**Results:**

We identified 19 consecutive patients for whom the needle tip of the TruePass™ suture passer was broken in the 437 shoulder surgeries. Based on the finite element analysis of Abaqus, around the tip and the notch of the intact needle was a large stress concentration. The average puncture force required by intact needle tip and the broken tip is 61.78N and 78.23N respectively. While the intact tip with notch is easier to break than the broken tip.

**Conclusions:**

The notch of the needle is a weak point in mechanics. The broken needle without the notch still has good tendon piercing and thread passing ability. The notch of needle may be not necessary, and the tip of the needle should be modified.

## Introduction

As arthroscopic technologies continue to develop, arthroscopic rotator cuff repair has become an alternative choice for orthopedic surgeons rather than the open surgical approach (1, 2). Arthroscopic rotator cuff repair has shown to accelerate postoperative recovery of shoulder joint motion (3), effectively alleviate shoulder pain (4, 5), improve dysfunction in cases of rotator cuff tear (6), and provide durable successful clinical results for more than 5 years (7). Arthroscopic rotator cuff repair performance has increased gradually. The narrow working space in the shoulder joint cavity and the importance of the position of sutures in cuff repair raised the demand for various specialized instruments for surgery (8). A suture passing device, as a kind of specialized instrument, combines a nitinol needle with a grasping device to grasp tissue and retrieve sutures in one step. This instrument reduces the surgery duration and facilitates the suturing process. The notch at the tip of the needle is designed to help the suture to penetrate the tendon along with the needle passage.

However, the notch at the tip of the needle often causes problems during the surgery. When the needle tip penetrates through thick tendon or the acromion in the narrow subacromial space, the needle is vulnerable to break at the notch site (9). Broken needle tips are tiny metal pieces, so that they cannot be observed easily during surgery and can only be identified by postoperative radiography. However, it is too late for the postoperative observation of broken needle tips, and reoperation is unacceptable for patients and may result in more serious injuries in patients, which is also a source of medical dispute. A few literature studies have discussed about the suture passer needle breakage issue recently (9–13). The phenomenon of a broken suture passer needle was first described by Song and Ramsey in a case report in 2008 (11). Although they founded that intratendinous needle remnants do not influence the range of motion of the shoulder 2 years postoperatively (11), clinical observations indicate that these remnants may be the source of pain in patients when they actively try to reach toward the upper back (11). Recently, Chung et al. pointed that needle remnants seemed to be associated with higher visual analog score in the early postoperative period (9). Therefore, the problem about broken suture passer needle remnants cannot be ignored and needs to be resolved.

Previous studies only reported the needle breakage issue without suggestion of a solution. We also observed such a phenomenon during the shoulder arthroscopic surgery. Additionally, we found that the reason that made us ignore the problem about broken needle was that the suturing process could be successfully achieved without influence from the broken needle. Therefore, we suggested whether the notch of the suture passer, a mechanically vulnerable position, could be modified and we further investigated whether snapping the needle at the notch in advance would influence the suture failure rate of arthroscopic rotator cuff repair. Finite element analysis (FEA) on the needle fracture was used to explore the stress distribution difference between the needles with and without snapping in advance. We hypothesized that the needle with snapping at the notch in advance would result in a reduced occurrence of needle breakage than the needle without snapping in advance.

## Methods and materials

### Clinical study

**Figure 1:**
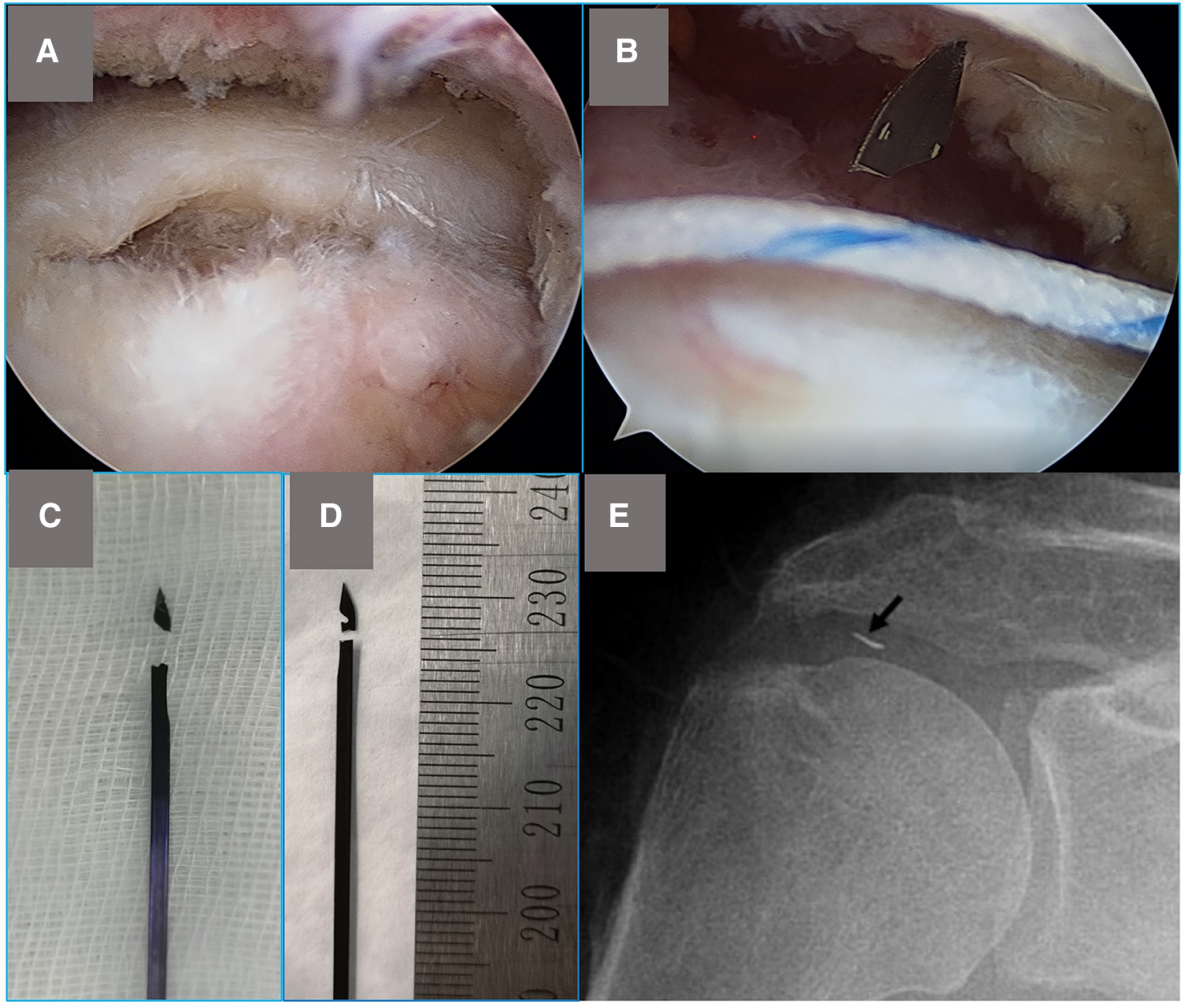
(**A**) Female, 59 years old, a full-thickness tear of the supraspinatus tendon was observed from the posterolateral approach. (**B**) The suture needle tip was broken during the operation, and the broken needle tip was found in the subacromial space. (**C**) Photograph of the broken needle of the TruePass™ suture passer, used in surgery (**B**). (**D**) Photograph of a needle of the TruePass™ suture passer snapped in advance. The break is located at the indentation in the needle. (**E**) Postoperative anteroposterior radiograph of a woman, 72 years old, 1 week after arthroscopic rotator cuff repair. The arrow points to a metallic foreign body that represents the broken tip of a suture passing device.

From 2017 to 2021, 426 consecutive patients, 175 men and 251 women, aged 23–83 years, mean age 53.2 ± 11.6 years, 437 shoulders (11 cases were bilateral), underwent arthroscopic repair for full-thickness rotator cuff tear at the authors’ institution. The Institutional Review Board of the Shanghai Jiaotong University School of Medicine Renji Hospital approved the study protocol (No. KY2020–177) and granted exemption for patient consent. The indications for surgery included shoulder pain or disability refractory to supervised nonsurgical treatment, including medication, local steroid injections, and physical therapy program, for a minimum of 6 months. Patients were excluded when they had other pathologies, like shoulder instability or chondral procedures. All procedures were performed by one shoulder-trained surgeon group with the TruePass™ suture passer (Smith and Nephew, United States). The broken parts of the needles, the tips of broken needle where were removed and whether it is left during the operation were recorded (Figure 1).

### Needle FEA

#### Model and material properties

**Figure 2:**
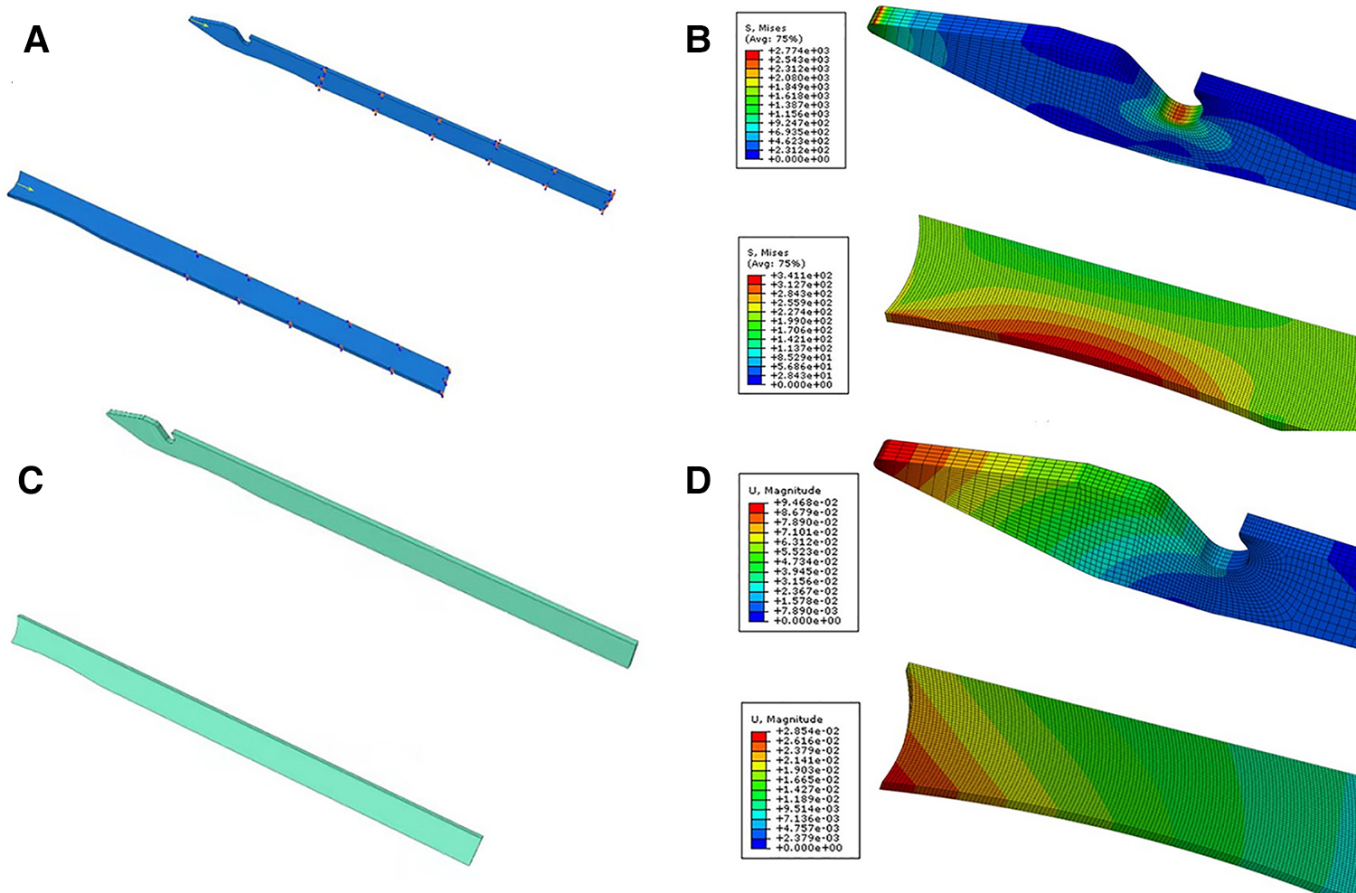
The needle models and their respective boundary conditions and loads. The model of the intact needle or the needle without notch (**A**). Boundary conditions and loads of the intact needle or the needle without notch (**B**). Distribution of the von Mises stresses in the models; the intact needle or the needle without notch (**C**). Distribution of the deformation in the models; the intact needle or the needle without notch (**D**).

Since the stress on the tip of the intact needle was mainly analyzed, SolidWorks (Version 2018, Dassault, France) software was used to create a model of the anterior part of the intact needle (Figure 2A). A three-dimensional model of the broken needle was created by removing the head from the notch of the needle (Figure 2B). The models were stored in IGS format and imported into Abaqus (version 6.14–1, Dassault, France) software for finite element analysis. The needle was made of titanium alloy. The models were considered homogeneous and continuous medium, elastic and isotropic, and their Young's modulus and Poisson's ratio were 110 GPa and 0.34, respectively. In Abaqus software, the models were given a hexahedron quadratic reduction integral unit (C3D20R) after proper component segmentation.

#### Boundary and loading condition

The posterior end of the intact needle was completely fixed (U1 = U2 = U3 = UR1 = UR2 = UR3 = 0), and a 100 N force was applied on the tip of the needle in the direction of the needle body (Figure 1C). The back end of the broken needle was also completely fixed (U1 = U2 = U3 = UR1 = UR2 = UR3 = 0), and 100 N load was applied to the concave part of the front end of the broken needle in the direction of the needle body (Figure 2D). In order to ensure the accuracy of stress and strain of the models, experiments on models with different mesh densities were carried out.

### Mechanical test

**Figure 3:**
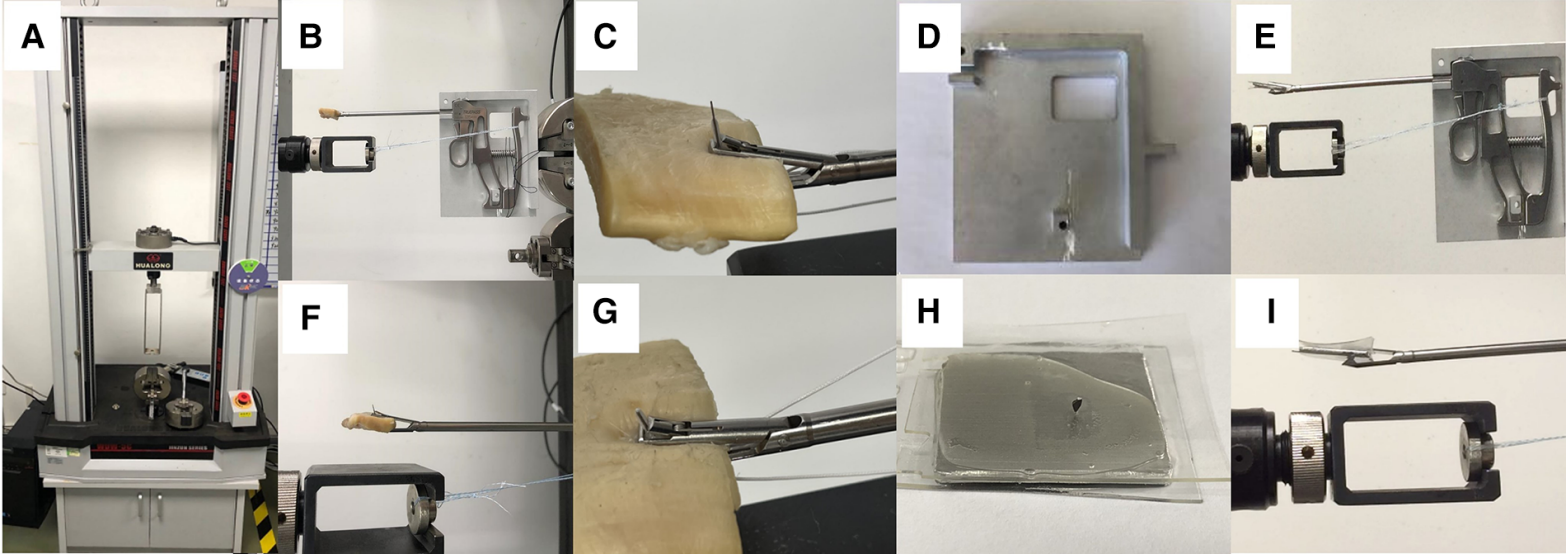
The electronic universal mechanical testing machine (**A**), puncture performance test (**B, F**), loaded puncture performance test (**C, G**), holding tool (**D**), TruePass™ suture passer assembled on the holding tool (**E**), the metal impact plate (**H**) metal impact plate (**H**), TruePass™ suture passer assembled on the holding tool is fired to make the needle core puncture the metal impact plate. Figure (**I**) is a partial magnification of figure (**E**).

The broken needle models were uniformly prepared with one surgical scissor to break the intact needle at 1 mini meter proximal to the needle's notch position in advance. The intact needle model and broken needle model are shown in Figure 3. In this experiment, five pieces of TruePass™ disposable needle with intact tips and five broken tips were selected. After the reassembly of the TruePass™ and disposable needles, the metal impact plate is clamped at the TruePass™ head, and then a stable and recordable traction force is transferred to the TruePass™ thread connector and TruePass™ disposable special suture needle in two states through the electronic universal mechanical testing machine (WDW-5C, Shanghai Hualong Testing Instrument Co., Ltd.), so as to simulate the metal impact plate and simulate the arthroscopic hand. During the operation, the actual use of the suture needle hitting the hard object during the operation and the required experimental data were recorded by the experimental equipment. Each suture needle is excited for several times. Once the fracture occurs, the batch experiment is stopped, and the minimum value of the batch experiment record is taken.

#### Puncture performance test

Five TruePass™ disposable special suture needles with intact needle tips and five broken needle tips were selected. First, the TruePass™ thread passing device and TruePass™ disposable special suture needle are assembled, and then the fresh bovine tendon, 7.5 mm thick, is clamped at TruePass™. Then, the TruePass™ thread connector and TruePass™ test tool are installed on the electronic universal mechanical testing machine (WDW-5C, Shanghai Hualong Testing Instrument Co., Ltd.). A stable and recordable traction force is transmitted to TruePass™ through the cooperation of the electronic universal tester and TruePass™ testing and trial tooling. In order to simulate the actual use of a suture needle in the human body during arthroscopic surgery, the TruePass™ disposable special suture needle under two conditions (intact needle tip/broken needle tip) was excited to conduct the puncture performance test on fresh bovine tendon (Figure 3), and the required experimental data were recorded by the electronic universal mechanical testing machine (WDW-5C, Shanghai Hualong Testing Instrument Co., Ltd.). Each suture needle is excited five times, and the recorded value of the first two times is taken.

#### Loaded puncture performance test

Load puncture performance test in this experiment, five TruePass™ disposable special suture needles with intact needle tips and five broken needle tips were selected, as well as, ultrahigh molecular weight polyethylene (HOCORE, 2#, Shanghai BJ-KMC Medical Technology Co., Ltd.). First, the TruePass™ thread passing device and TruePass™ disposable special suture needle are assembled with ultrahigh molecular weight polyethylene (HOCORE, 2#), and then the fresh bovine tendon, 4 mm thick, is clamped at TruePass™. Then, the TruePass™ thread connector and TruePass™ test tooling are installed on the electronic universal mechanical testing machine (WDW-5C, Shanghai Hualong Testing Instrument Co., Ltd.). A stable and recordable traction force is transmitted to TruePass™ through the cooperation of the electronic universal tester and TruePass™ testing and trial tooling. In order to simulate the actual use of a suture needle in the human body during arthroscopic surgery, the TruePass™ disposable special suture needle under two conditions (intact needle tip/broken needle tip) was excited to conduct the puncture performance test on fresh bovine tendon (Figure 3), and the required experimental data were recorded by the electronic universal mechanical testing machine (WDW-5C, Shanghai Hualong Testing Instrument Co., Ltd.). Each suture needle is excited five times, and the recorded value of the first two times is taken. For each loaded suture puncture, the successful suture crossing shall prevail, and whether the suture crossing is successful shall be recorded.

### Statistical analysis

Continuous variables were expressed as means standard deviations (SD). In order to compare the difference between the two groups in finite element analysis of Abaqus and mechanical test, independent sample *t*-tests were used. The software IBM SPSS statistics 24.0 (SPSS Inc., Chicago, IL, United States) was used for all statistical analyses, and *p* < 0.05 was considered statistically significant.

## Results

### Rate of needle breakage of antegrade suture passer

In this study, we identified 19 consecutive patients who had the incident that the needle tip of TruePass™ suture passer was broken. Eighteen needle tips were removed during surgery without further tissue injury. One needle tip was removed in the second surgery. One needle tip was embedded inside the tendon and hard to be removed, to avoid further injury to tendon tissue, we did not remove it from the tendon with agreement of patient. The patient with the remnant needle tip was followed up for 3 years without any complaints. In total, 19 broken needles, 18 tips removed were measured, and needle tips were found in different places (Figure 1 and Table 1). In addition, we unexpectedly found that rotator cuff suture can be carried out with a broken needle. The location of breakpoints on broken needles was measured. Fifteen needles were broken in the groove and three needles were broken within 3 mm proximal of the groove.

**Table 1:** Where were the tips found?

	In joint	In tendon	In subacromial space
Number of tips	3	2	14
Took out in first surgery	2	1	14
Took out in second surgery	1	0	0
Remnant	0	1	0

### Finite element analysis of Abaqus

Based on the finite element analysis of Abaqus, the stress cloud diagram obtained is shown in Figure 2. As can be seen, around the tip and the notch of the intact needle, there was a large stress concentration, with a maximum von Mises stress of 2774 MPa around the tip and 2435 MPa around the notch, and the failure form was the collapse of the tip and around the notch fracture. On the contrary, the stress distribution of the broken needle was good, with no obvious stress concentration, and the maximum the von Mises stress was 341 MPa.

The difference in the behavior between the intact needle and the broken needle is illustrated in Figure 2, which shows the displacement of the models subjected to 100 N load. It can be found that the overall displacement amplitude of the surgical needle varies between 0 mm and 9.468 × 10^−2^ mm, while the overall displacement amplitude of the broken needle varies from 0 mm to 2.854 × 10^−2^ mm, with obvious differences ( *р* <0.05).

### Mechanical

**Figure 4:**
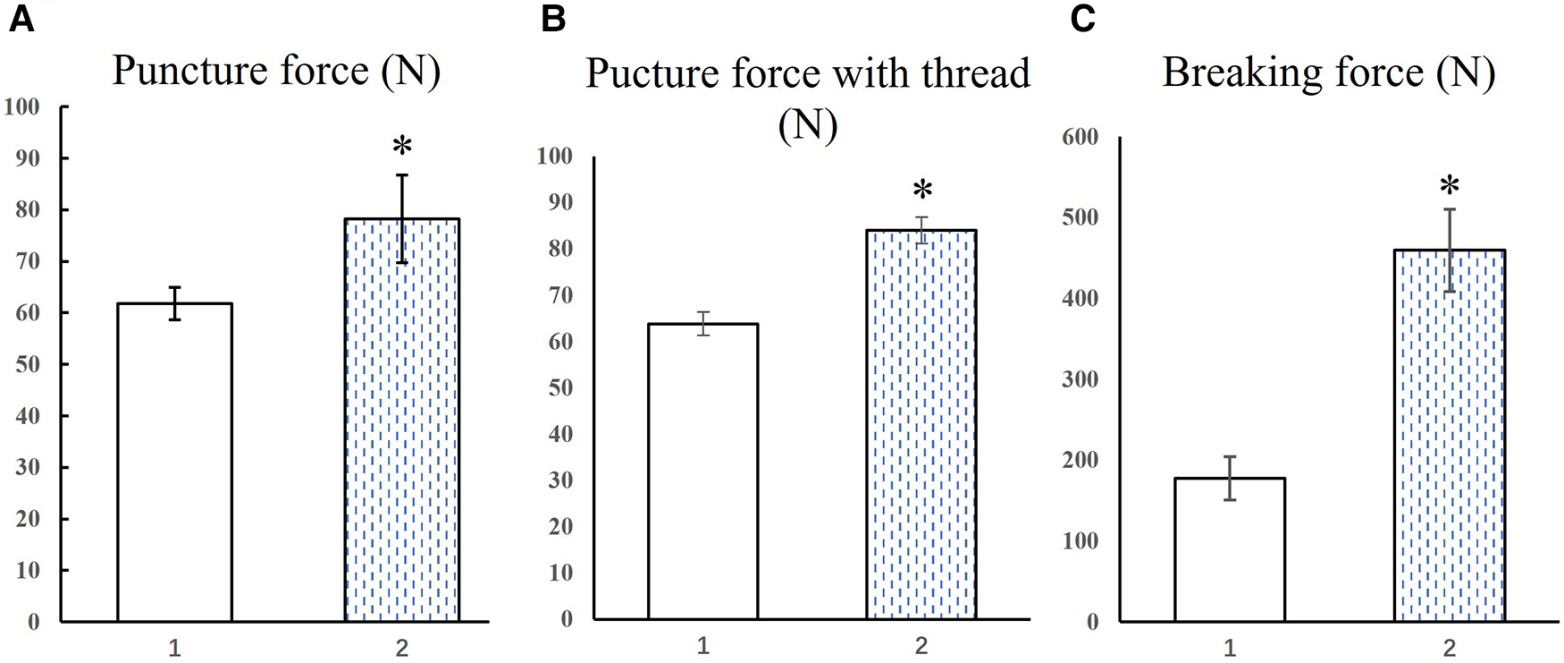
Needle with intact tip (1), needle with broken tip (2), *р < 0.05.

The force values required by TruePass™ disposable special suture needle under two states were collected. There were two groups of data collected, with 10 values in each group (Figure 4). According to the data of puncture force collected from the above two groups, the average value of puncture force required by TruePass™ disposable special suture needle with intact needle tip is about 61.78 (±3.18) N, while that of TruePass™ disposable special suture needle with broken tip is about 78.23 (±8.51) N, the difference was statistically significant (*р* < 0.05), (Figure 4).

The force values of TruePass™ disposable special suture needle under two conditions were collected. There were two groups of data collected, with five values in each group (Figure 4). Note that, in the experiment, the needle tip was obviously deformed but not broken after impacting the metal impact plate. Due to the preciseness of the experiment, the needle with deformed tip is judged as broken. The average breaking force of the TruePass™ disposable special suture needle with an intact tip is about 183.27 (±26.5) N, while that of TruePass™ disposable special suture needle with broken tip is about 457.37 (±50.9) N, the difference was statistically significant (*р* < 0.05) (Figure 4).

## Discussion

A suture passer facilitates sutures to penetrate through soft tissues such as tendons, and the needle tip received loading from the surrounding tissues. The total stress can lead to breakage of the needle at the notch or near the notch. Therefore, the optimization of needle tip is important. Analysis of distribution in the needle tip is an essential step, as maximum stress in the needle determines the success or failure of arthroscopic rotator cuff repair (14). Excessive stress can lead to needle tip breakage. In our cases, we found that most broken needle tips were found in the subacromial space, and only a few were found in the articular cavity or in the tendon tissue. However, due to the small sample size and other problems, there is no statistical analysis.

We also found that the most easily broken part of the device is at the indentation in the needle tip through FEA simulation. The unique memory function and flexibility of this nickel-titanium alloy allow the surgical needle return to its original shape after bending. This alloy has been widely used in clinical applications. The mechanical fracture of nickel-titanium alloy has been a hot issue in the endodontology field, and the phenomenon of needle breakage in arthroscopic rotator cuff repair has been reported too (9, 11, 15). Our trial provides additional support to previous studies (9, 11). Although we used a TruePass™ suture passer in this study, the same result would be observed with the use of a FastPass™ Scorpion suture passer (Arthrex, Naples, FL, United States).

The notch at the needle tip is designed to aid the passage of sutures through tissue. However, suture passers use the same disposable needle frequently during a single case; repeated loadings on the needle tip may easily lead to breakage of the needle at the notch. What we found in this experiment might solve this problem. It should be noted that the length of the needle breakage in advance should not exceed 2 mm proximal to the notch position. In clinical use, there are many factors affecting the stress distribution of the needle tip. The stress distribution is different due to different geometrical parameters, thus affecting the sensitivity of the bending fracture (15). Therefore, we could suggest medical device companies to use a flathead pin to replace the current needle tip. Of course, other puncture tools can also be used (16). Needle breakage may also be avoided with the progress of science and technology; new tools and methods should appear, including automated-assisted suture passage (17, 18).

The needle should be marked by scales so that the broken tip can be found in a timely manner. We also have some advice for surgeons. First, when stitching, the surgeon should avoid stretching and pulling of the tendon. Thus, moderate force should be used to suture thick and tough tendon tissues (14). If the rotator cuff tissue for suture is thick, or accompanied by narrow subacromial space, and the needle tip is easy to contact the bone on the subacromial surface, the operator should be careful to avoid needle breakage (14).

In addition, the subacromial space is very narrow, which results in easy needle breakage when needle tips touch the hard bone (19, 20). It is necessary to check the distal part of the suture passer arthroscopically to avoid this problem. The probability of needle breakage may be reduced if the acromion molding is completed first and the prominent Neer's type III acromion osteophyte is removed in patients with a narrow subacromial space (21, 22). Most broken needle tips are found in the articular cavity or subacromial space and rarely in the tendon tissues. They can generally be removed using a thread grabber arthroscopically. Sometimes we need c-arm fluoroscopy to find the broken tip. In addition to that, stitching needles need to be disposable or replaced regularly. While these results suggest an association between the distribution of stress and needle tip breakage, the present study still has a limitation. We lacked a strict multicenter clinical trial to prove our conclusion from this trial.

## Conclusions

Through clinical research, mechanical testing, and finite element analysis, we found that the notch of the needle is a weak point in mechanics, and breakage often occurs in or near this point. At the same time, the broken needle without a notch still has good tendon piercing and thread passing ability. The study highlights that all devices should be carefully checked during and after surgery. The notch of needle may be not necessary, and the tip of needle should be modified. The investigation provides knowledge to assist in achieving better future designs.

## Data Availability

The original contributions presented in the study are included in the article/Supplementary Material, further inquiries can be directed to the corresponding authors.
